# The Extensor Response (Babinski's Sign)

**Published:** 1916-06-24

**Authors:** 


### THE EXTENSOR RESPONSE (BABINSKI'S SIGN).

### Its Positive and Negative Value.

The description of this sign in 1898 was a welcome advance in clinical knowledge, seeing that it entered as a confident announcement in a region often subject to debate and uncertainty. Here, at last, was an infallible means of distinguishing between organic and functional nervous disease; or, if this is to put the case too high, here was a sign in the presence of which organic change could be confidently asserted, allowing that its absence did not necessarily mean a diagnosis of mere functional disturbance. This claim has now been subjected to the test of years, and its validity is almost universally admitted. If exceptions have been described, they have been so few that they hardly merit attention. It is true that some records have announced the existence of an extensor response together with other nervous symptoms, and the subsequent disappearance both of one and the other. But to conclude that cases with this history are purely functional is a severe demand to make. Surely it must be admitted that there may be physical disturbances in the central nervous system which are not beyond the possibility of recovery. Babinski himself thought so, and in his original paper he plainly says that his sign may be present in cases of paralysis which prove to be curable. On the whole, it may confidently be affirmed that in a debate between organic and functional disease the presence of an extensor response in one or both feet definitely settles the issue in favour of organic disease. It is, therefore, an observation of very high clinical value.

If, however, functional nervous disease can be excluded as an explanation of Babinski's sign, there are certain physiological and quasi-pathological conditions of which the same cannot be said. They may be briefly mentioned here, and the greater number of them, it will be seen, hardly propose any clinical issue of importance. Thus an extensor response is common in infants up to about two years of age, and this especially during sleep; even in sleeping adults it may be detected, and it is usual as a temporary condition after an epileptic fit; the administration of strychnine may produce it, and the same, it is said, is true of hyoscine. All these observations are interesting, and have some measure of importance, but they scarcely limit the value of Babinski's sign as a proof of organic disease. So long as they are remembered they cannot lead to any diagnostic error.

Speaking broadly, there are two clinical conditions in which the value of Babinski's sign reaches its highest level. The one is where evidences of nervous disease, though abundant, are capable of being explained as manifestations of hysterical or functional disturbance. The other is where such evidences are few, and a doubt may arise whether disease of any kind does or does not exist.

Take the first of these positions. It is a recognised experience to find such symptoms as hemiplegia, and perhaps hemianæsthesia, associated with exaggeration of the tendon jerks, and sometimes (though rarely) with ankle clonus, and yet to learn that, sooner or later, the patient makes a complete recovery, though the course of this may be marked by one or several relapses. In such circumstances the early problem is to be sure whether there is or is not organic disease. Motor paralysis or spasm will not help. Neither will anæsthesia, though the more conspicuous this is the more likely is the case to be of the functional order. Again, conspicuous knee-jerks and heel-jerks are just as likely to be due to functional disturbance as to organic change. About ankle clonus a more guarded proposition is necessary, for a sustained and rhythmical clonus is rare apart from organic change; still, it is occasionally noted in functional disease, and, therefore, its message is not an absolute one. It is here that an extensor response leaves no room for uncertainty. Whatever may be said of the possible meaning of other signs, there is but one meaning possible to Babinski's sign—namely, the presence of an organic lesion. Unfortunately its absence does not necessarily mean functional disease, for not all organic changes produce it. This is a serious limitation on the negative value of the test, but on its positive side it remains significant.

Another position showing the value of the extensor response is where evidences of nervous disease are so few and slight that their existence may be doubted or overlooked. Two instances may be quoted. In the one a man, middle-aged or elderly, has some kind of an attack associated, perhaps, with giddiness or mental confusion; the condition is a temporary one, and is placed, perhaps, to the fault of the stomach or to the discredit of the liver. Yet on examination one of the plantar reflexes gives an extensor response. The conclusion is certain that what was regarded in all probability as a mere digestive disturbance was really a limited cerebral hæmorrhage or thrombosis. Though not in form, the case in essence is one of hemiplegia in which the lesion is so limited that it has produced no evidence of its existence other than a unilateral extensor response. Obviously the effect on diagnosis and prognosis can hardly be exaggerated, and this true conception of the situation is brought about entirely by the observation of Babinski's sign. In another class of cases a young adult, often a woman, complains that she gets very tired on walking, or she has numbness or tingling sensations, or she drops objects from her hands, or there is some bladder disturbance, or there is an exhibition of hysteria in the popular sense of this term. Such cases are apt to be dismissed as needing a "change" or a "tonic," and to receive soothing assurances in reference to the future. But every now and again examination will detect an extensor response, and it becomes morally certain that the symptoms are early manifestations of disseminated sclerosis. Here, again, there is a region of experience in which the positive value of Babinski's sign is exhibited. That it is not more widely appreciated simply means that it is not looked for often enough.

